# Meliponini Geopropolis Extracts Induce ROS Production and Death in *Leishmania amazonensis* Promastigotes and Axenic Amastigotes In Vitro

**DOI:** 10.3390/biology14020162

**Published:** 2025-02-06

**Authors:** Kamila M. Sette, Andreza R. Garcia, Luzineide W. Tinoco, Anderson S. Pinheiro, Igor A. Rodrigues

**Affiliations:** 1 Programa de Pós Graduação em Ciências Farmacêuticas, Faculdade de Farmácia, Universidade Federal do Rio de Janeiro, Rio de Janeiro 21941-902, RJ, Brazil; kamila.sette@hotmail.com (K.M.S.); raposo.arg@gmail.com (A.R.G.); 2 Laboratório Multiusuário de Análises por RMN, Instituto de Pesquisa de Produtos Naturais, Universidade Federal do Rio de Janeiro, Rio de Janeiro 21941-902, RJ, Brazil; luzitinoco@gmail.com; 3 Laboratório de Bioquímica Molecular, Departamento de Bioquímica, Instituto de Química, Universidade Federal do Rio de Janeiro, Rio de Janeiro 21941-902, RJ, Brazil; pinheiro@iq.ufrj.br; 4 Laboratório de Investigação de Substâncias Bioativas, Departamento de Produtos Naturais, Faculdade de Farmácia, Universidade Federal do Rio de Janeiro, Rio de Janeiro 21941-902, RJ, Brazil

**Keywords:** native bee products, leishmanicidal activity, mitochondrial activity, human cutaneous leishmaniasis

## Abstract

Leishmania amazonensis, a cause of cutaneous leishmaniasis in Brazil, is a neglected disease with toxic and inconsistently effective treatments. The parasite’s survival depends on managing oxidative stress, making redox-regulating enzymes potential therapeutic targets. Geopropolis, a resinous product from native stingless bees, shows promising antiparasitic effects. This study aims to evaluate the anti-*L. amazonensis* activity of geopropolis produced by *Melipona bicolor*, *M. marginara*, *M. mondury*, and *M. quadrifasciata* (two samples), targeting enzymes responsible for the parasite’s redox balance. Ethanol extracts of geopropolis produced by each bee (BCRL, MRGT, MNDY, MNDA(1), and MNDA(2), respectively) were analyzed for total phenolics and flavonoids. Promastigotes and axenic amastigotes were treated with various extract concentrations, and parasite viability was assessed using the resazurin reduction method. Cytotoxicity was tested on peritoneal macrophages, RAW 264.7, VERO cell lines (MTT assay), and erythrocytes (hemolysis assay). Additionally, mitochondrial dehydrogenase activity, reactive oxygen species (ROS) production, the inhibition of recombinant arginase, and autophagic activity were also evaluated in treated parasites. MRGT showed the highest levels of phenolics (762 mg GAE/g) and flavonoids (345 mg QE/g). MDRY was more effective against promastigote and axenic amastigote forms (IC_50_ = 168 and 19.7 µg/mL, respectively). MRGT showed lower cytotoxicity against RAW 264.7 and VERO (CC_50_ = 654 µg/mL and 981 µg/mL, respectively). Erythrocytes exhibited reduced sensitivity to MNDA(2) (HC_50_ = 710 µg/mL). The activity of dehydrogenases and *Li*ARG was reduced by treating the parasites with the extracts following the induction of ROS and autophagic activity. These results highlight geopropolis extracts as a source of substances with anti-*L. amazonensis* activity capable of inducing oxidative stress on the parasite.

## Simple Summary

*Leishmania amazonensis*, the causative agent of cutaneous leishmaniasis in Brazil, is notoriously difficult to treat due to the toxicity and inconsistent efficacy of current therapies. This disease causes skin and mucosal lesions, often leading to disfigurement and social stigmatization. Our study explored the pharmacological potential of geopropolis, a resinous product from stingless bees, as a natural antileishmanial treatment. Geopropolis extracts from *Melipona bicolor*, *Melipona marginata*, *Melipona mondury*, and *Melipona quadrifasciata* were evaluated for their effects on *L. amazonensis* and cytotoxicity against mammalian cells. The extracts demonstrated notable levels of phenolics and flavonoids, known for their antioxidant properties, which could aid lesion healing. A promising antileishmanial activity was observed, particularly in the geopropolis extract from *M. mondury*. All the extracts induced oxidative stress in the parasite by increasing reactive oxygen species (ROS), targeting its critical redox balance and survival mechanisms. In addition, the extracts were more toxic to parasites than mammalian cells. These findings position geopropolis as a natural source of bioactive compounds with the potential to contribute to the development of safer, more effective treatments for cutaneous leishmaniasis. This approach could significantly advance the fight against this neglected disease while addressing its social and medical challenges.

## 1. Introduction

Leishmaniasis is a neglected tropical disease caused by infection with *Leishmania* parasites, transmitted through the bite of infected female phlebotomine sandflies. Around 20 species of *Leishmania* are capable of infecting mammals and causing the disease. According to the World Health Organization (WHO), it is estimated that over 1 billion people live in endemic areas and are at risk of infection. The cutaneous form is the most prevalent, affecting an estimated 600,000 to 1 million people worldwide annually, though only about 200,000 cases are officially reported to the WHO [1]. In the Americas, there are several cutaneous manifestations of the disease known as American Tegumentary Leishmaniasis (ATL), which includes cutaneous leishmaniasis (CL), mucocutaneous leishmaniasis (MCL), disseminated cutaneous leishmaniasis (DCL), and diffuse cutaneous leishmaniasis (DL). Variations in the clinical manifestations of the disease also reflect a complex immune response of the host and parasite virulence factors [2]. In Brazil, ATL is predominantly caused by the species *Leishmania (Viannia) braziliensis*, *L. (V.) guyanensis*, and *L. (Leishmania) amazonensis* [3]. In recent years, an increase in ATL cases has been observed across Brazilian territory, with significant epidemic outbreaks resulting from devastating urbanization processes and the occupation of previously vegetated areas, especially in the southeast, northeast, and north regions.

The *Leishmania* parasite has evolved efficiently and can survive the oxidative stress generated by the host cell through various defense mechanisms [4]. Some lower eukaryotes, like *Leishmania*, also have a unique L-arginine metabolism pathway for the biosynthesis of polyamines and trypanothione. The hydrolysis of L-arginine by arginase (ARG) leads to the production of L-ornithine and urea in the first step of polyamine biosynthesis. Spermidine, the end product of the polyamine pathway, combines with glutathione (GSH) to synthesize trypanothione (T(SH)_2_). This molecule plays a key role in maintaining the thiol redox balance, synthesizing deoxyribonucleotides, enabling drug resistance, and defending against chemical and oxidative stress by neutralizing reactive oxygen and nitrogen species produced by host cells. Inhibiting *Leishmania* ARG can reduce T(SH)_2_ production, weakening the parasite’s defenses against oxidative stress [5]. Moreover, an increase in reactive oxygen species (ROS) can lead the parasite to death by an apoptotic process [6].

The induction of oxidative stress is a promising strategy to eliminate *Leishmania*, which has already been demonstrated as a mode of action for current antileishmanial drugs. Pentavalent antimonials can induce oxidative stress in parasites by inducing an efflux of intracellular trypanothione and glutathione, along with trypanothione reductase inhibition [7,8]. The treatment of the disease includes the use of pentavalent antimonials (meglumine antimoniate and sodium stibogluconate), amphotericin B deoxycholate, liposomal amphotericin B, pentamidine isethionate, paromomycin sulfate, and miltefosine [9]. Generally, the chemotherapy used to treat ATL has associated challenges, limiting its use due to (i) high cost, (ii) high toxicity, (iii) variability in treatment efficacy, (iv) numerous adverse effects, and (v) the emergence of resistant strains [10]. No vaccines are available against human leishmaniasis [11], contributing to disease dissemination.

Meliponines, or stingless bees, represent the largest eusocial bees worldwide. In Brazil, approximately 200 species across 29 genera are distributed throughout the country, with 89 species being endemic [12]. These bees belong to the Apidae family and the Meliponinae subfamily, further divided into two tribes: *Meliponini* and *Trigonini*. Meliponines differ from honeybees (*Apis mellifera*, Apidae) in several ways, with a notable distinction being sting morphology: female meliponines either lack a sting entirely or have an atrophied one [13,14]. The ecological significance of meliponines is undeniable, as these insects are natural pollinators of native plants across various biomes and play an important socioeconomic role, being used in the pollination of several crops and the production of honey, pollen, and propolis or geopropolis [15].

Propolis is a viscous product made by mixing bee secretions (saliva and wax) with plant resins, and its antimicrobial properties provide a chemical defense for the bees and their honey against microbial action [16]. Both honeybees and several species of meliponines can produce propolis. Geopropolis, a resin enriched with soil or clay, differs from propolis primarily due to its mineral content. The mineral content of geopropolis has been investigated as a traceability parameter, as it can reflect the composition of the soil where it is produced. Additionally, it has been recognized for its nutritional value as a source of essential elements, including Ca, Cu, Fe, K, Mg, Mn, P, and Zn [17]. Geopropolis is produced by certain species of stingless bees, particularly those belonging to the *Meliponini* tribe. Despite these differences, geopropolis serves a similar function, protecting the beehive from invaders, including other insects and microorganisms [18,19]. In addition, geopropolis is widely used in traditional medicine by various populations for its wound-healing, gastroprotective, and antibiotic properties [20].

Geopropolis comprises approximately 50% resins, 30% wax, 10% essential oils, 5% pollen, and 5% other organic compounds associated with the inorganic compounds [21]. Various biological activities of geopropolis have been investigated and described in the literature, including antibacterial, antioxidant, anti-inflammatory, immunomodulatory, anticancer, and antimicrobial activities [21,22,23,24,25,26,27]. Most of the bioactivity of geopropolis samples has been attributed to their phenolic and terpenoid contents [18,28]. Notably, the activity of geopropolis against human protozoan parasites remains underexplored. Here, we evaluated the anti-*L. amazonensis* activity of geopropolis produced by the species *Melipona bicolor*, *M. marginata*, *M. mondury*, and *M. quadrifasciata*, targeting the parasite’s redox metabolism.

## 2. Materials and Methods

### 2.1. Chemicals and Culture Media

2,2′-Azobis(2-methylpropionamidine) dihydrochloride (AAPH), Deuterium dimethyl sulfoxide (DMSO-d_6_), 2′,7′-dichlorodihydrofluorescein diacetate (H2DCFDA), dimethyl sulfoxide (DMSO), Dulbecco’s Modified Eagle’s Medium (DMEM), monodansylcadaverine (MDA), gallic acid, Grace’s medium, 10,000 U/mL penicillin and 10 mg/mL streptomycin solution (10 mL of this solution per liter of medium for cell cultures), antimony potassium tartrate trihydrate (SbIII), quercetin, resazurin, Schneider’s medium, tetrazolium salt (MTT), and 3-(trimethylsilyl)-1-propane sulfonic acid sodium salt (DSS) were purchased from Sigma-Aldrich (St. Louis, MO, USA). Fetal bovine serum (FBS) was purchased from LGC Biotecnologia (Cotia, SP, Brazil).

### 2.2. Collection of Geopropolis and Extraction Process

Geopropolis from *Melipona quadrifasciata* Lep. (1), *Melipona mondury* Smith, 1836, *Melipona bicolor* Lep., and *Melipona marginata* Lep. were collected in Guapimirim (RJ, Brazil) in January 2021. A fifth sample, produced by *M. quadrifasciata* (2), was collected in Rio de Janeiro city (RJ, Brazil) in April 2019. The samples were kindly provided by stingless beekeepers of the *Associação de Meliponicultores do Estado do Rio de Janeiro* (AME-Rio). This study was duly registered in the National System for the Management of Genetic Heritage and Associated Traditional Knowledge (registration code A6DAB60).

The crude geopropolis samples (200 g) were subjected to static maceration in 800 mL of 95% ethanol for 24 h in the dark. Ethanol was selected for extraction procedures due to its current use in propolis and geopropolis formulations for human consumption. The solid phase was removed by filtration, and the liquid phase was stored overnight at −20 °C to precipitate wax. Afterward, the liquid phase was filtered and stored again at −20 °C, repeating the process until no further wax precipitation occurred. Finally, the liquid phase was dried using a rotary evaporator, yielding the following extracts: MNDA(1) and MNDA(2) (from *M. quadrifasciata*), with yields of 2.8% and 4.5%, respectively; BCLR (from *M. bicolor*), 22%; MRGT (from *M. marginata*), 1.12%; and MDRY (from *M. mondury*), 1.28%. These extracts were prepared as stock solutions by diluting them in DMSO at a concentration of 100 mg/mL. For biological assays, the final DMSO concentration did not exceed 1%.

### 2.3. Cell Culture

Promastigote forms of *L. amazonensis* (IFLA/BR/1967/PH8) were obtained from the *Coleção de Leishmania do Instituto Oswaldo Cruz* (CLIOC/FIOCRUZ, RJ, Brazil) and maintained at 26 °C in Schneider’s culture medium supplemented with 10% fetal bovine serum (FBS). Axenic amastigote forms were obtained by differentiating promastigote forms in an acidified Grace’s medium (pH 5.3) at 32 °C, as previously described by Garcia et al. (2023) [29]. Cell differentiation was confirmed by optical microscopy.

RAW 264.7 and VERO cell lines were obtained from the *Banco de Células do Rio de Janeiro* (BCRJ/INMETRO) and maintained every 48–72 h in DMEM supplemented with 10% FBS and penicillin (100 U/mL)/streptomycin (100 µg/mL) at 37 °C in a 5% CO_2_ atmosphere.

Primary macrophages (MØ) were obtained by a peritoneal lavage of female BALB/c mice (6–8 weeks), previously stimulated with 0.5 mL of 3% thioglycolate solution. After 96 h, the animals were euthanized according to institutional policies (ethical approval 122/19—CEUA/UFRJ), and peritoneal lavage was performed with cold phosphate saline buffer (PBS, pH 7.4). The cells were centrifuged at 1700× *g* for 5 min, washed twice with cold PBS, and resuspended in a complete DMEM to a final density of 10^6^ cells/mL. The number of cells was determined using a Neubauer chamber. Finally, the cells were distributed into 96-well microplates (10^5^ cells/well) and incubated overnight prior to the biological assay.

### 2.4. Determination of Phenolic Content

#### 2.4.1. Total Phenolic Content

The total phenolic content (TPC) of the geopropolis extracts was determined using the microtiter Folin–Ciocalteu method [30]. In a 96-well microplate, 20 μL of geopropolis working solution (1 mg/mL) was mixed with 20 μL of Folin–Ciocalteu reagent. After 5 min, 20 μL of a 0.01 M Na_2_CO_3_ solution was added, followed by 125 μL of distilled water after an additional 5 min. Absorbance was measured at 750 nm using a microplate reader (SpectraMax i3x, Molecular Devices, Sunnyvale, CA, USA). A calibration curve was prepared using gallic acid as the standard (1.8–500 μg/mL), and the results were expressed as mg of gallic acid equivalents (GAE) per g of extract.

#### 2.4.2. Total Flavonoid Content

The total flavonoid content (TFC) of the geopropolis extracts was determined using the aluminum–flavonoid complexation assay [31]. In a 96-well microplate, 25 μL of geopropolis working solution was mixed with 100 μL of distilled water. Then, 10 μL of sodium nitrite (50 g/L) was added, and the mixture was allowed to stand for 5 min. Subsequently, 15 μL of aluminum chloride (100 g/L) was added, followed by 50 μL of 1 M NaOH and 50 μL of distilled water after 6 min. Absorbance was measured at 510 nm using a SpectraMax i3x reader (Molecular Devices, Sunnyvale, CA, USA). A calibration curve was constructed using quercetin as the standard (1.8–500 μg/mL), and the results were expressed as mg of quercetin equivalents (QE) per g of extract.

### 2.5. DPPH^•^ Scavenging Assay

The antioxidant capacity of the geopropolis extracts was evaluated using the DPPH^•^ scavenging assay [32]. For each sample, 20 μL of the working solutions (4 mg/mL for MNDA(1), MNDA(2), and MDRY; 0.5 mg/mL for BCRL; and 0.25 mg/mL for MRGT) were transferred to a 96-well microplate and mixed with 180 μL of DPPH solution (150 μM in 80% methanol). The mixture was incubated for 40 min in the dark. Absorbance was then measured at 515 nm using a microplate reader (SpectraMax i3x, Molecular Devices, CA, USA). The percentage of DPPH^•^ scavenging for each sample was calculated using the following equation: DPPH scavenging activity (%) = ((Abs_c_ − Abs_s_)/Abs_c_) × 100, where Abs_c_ = absorbance of the DPPH solution without the sample (control); Abs_s_ = absorbance of the DPPH solution with the sample.

### 2.6. Spectroscopic Analysis and Chemometric Approach

Nuclear Magnetic Resonance (NMR) spectroscopy was used to analyze the chemical profiles of the geopropolis extracts. The extracts (6 mg) were dissolved in 588 μL of a 0.05 mM DSS solution prepared in DMSO-d_6_. The samples were centrifuged at 11,000× *g* for 15 min, and 550 μL of each supernatant was transferred to 5 mm NMR tubes. One-dimensional ^1^H spectra were acquired using a Varian VNMRS500 spectrometer (Varian Inc., Palo Alto, CA, USA) with the following parameters: ^1^H, 499,78 MHz, 64K points, 1K accumulations, a spectral width of 20 ppm, a relaxation delay (d1) of 4.50 s, and a reference frequency (O1P) of 4.69 ppm.

The 1D ^1^H NMR spectra were processed using Mnova 15.0.0^®^ (Mestrelab Research, Santiago de Compostela, GA, ES). Phase and baseline alignment were adjusted, and DSS was calibrated as an internal standard with a chemical shift of 0.00 ppm. Peaks were normalized to the highest intensity peak, and a binning procedure was applied to generate 0.04 ppm buckets. Baseline regions at the beginning and end of the spectra were excluded from the analysis. The processed data were exported as a “.csv” file using Excel 2019^®^ (Microsoft Co, Seattle, WA, USA) and uploaded to the MetaboAnalyst 6.0 platform (https://www.metaboanalyst.ca/, accessed on 7 December 2024). Data normalization was performed using Pareto Scaling and multivariate analysis using the unsupervised learning algorithm Principal Component Analysis (PCA).

### 2.7. Antileishmanial Activity

#### 2.7.1. Leishmania Viability Assay

Promastigote forms (in the exponential phase) and axenic amastigote forms (recently differentiated) of *L. amazonensis* were prepared as described previously [29]. The antileishmanial assay was conducted using a microdilution technique followed by a viability assessment with resazurin. The number of promastigotes and axenic amastigotes was initially determined through direct counting using a Neubauer chamber. Subsequently, 10^6^ cells were seeded into a 96-well microplate containing increasing concentrations of geopropolis extracts (12.5, 25, 50, 100, 200, and 400 µg/mL) prepared in Schneider’s medium for promastigotes or Grace’s medium for amastigotes. Promastigotes and amastigotes were incubated at 28 °C and 32 °C, respectively, for 48 h. Untreated parasites served as the positive viability control, while parasites treated with trivalent antimony (SbIII) were used as the reference for antileishmanial activity. After incubation, 25 µL of 0.005% resazurin solution was added to each well to assess parasite viability [33]. The concentration required to inhibit 50% of parasite growth (IC_50_) for each extract was calculated using a nonlinear regression analysis of the dose–response curves generated with the data obtained above. Alternatively, the leishmanistatic or leishmanicidal effects of geopropolis extracts were evaluated using promastigote forms. The treatment procedure followed the same protocol described above, except that cultures were incubated at 28 °C for 168 h. The incubation period was chosen based on the growth curve of the *L. amazonensis* strain cultivated under our laboratory conditions. Parasite growth was monitored every 24 h by measuring turbidity via optical density (OD) at 600 nm using a microplate reader (SpectraMax i3x, Molecular Devices, CA, USA). The results were expressed as a percentage relative to the control, which was set at 100% growth [34]. A leishmanicidal effect was defined as the absence of cell growth throughout the incubation period. In contrast, a leishmanistatic effect was assigned to cases where inhibited cells resumed progressive growth over time.

#### 2.7.2. Mitochondrial Dehydrogenase Activity

Mitochondrial dehydrogenase activity was evaluated using the MTT assay described by Oliveira et al. (2023) with minor modifications [35]. Promastigote forms of *L. amazonensis* at a final density of 2 × 10^7^ parasites/mL were treated with geopropolis extracts (IC_50_ and 2×(IC_50_)) for 4, 12, 24, and 48 h. The number of cells was determined using a Neubauer chamber. After treatment, parasites were washed with PBS, counted using the trypan blue exclusion method [36], and resuspended at the same cell density in a final volume of 100 µL. Next, 40 µL of MTT solution (0.5 mg/mL in PBS) was added to each culture, which was then incubated for up to 4 h. Parasites were washed by centrifugation, and 100 µL of DMSO was added to dissolve the formazan crystals. Absorbance was measured at 570 nm using a microplate reader (SpectraMax i3x, Molecular Devices, Sunnyvale, CA, USA). Untreated parasites served as a negative control.

#### 2.7.3. Recombinant Arginase Activity

The amino acid sequence of ARG is highly conserved among pathogenic *Leishmania* species (>97%) [37], making the recombinant arginase from *L. infantum* (*Li*ARG) a suitable model for inhibitor screening. The procedures for enzyme expression and purification were previously described [38]. Enzyme inhibition assays were performed by incubating *Li*ARG (50 μg/mL) with 50 mM L-arginine in 50 mM CHES buffer (pH 9.5) in the presence of various extract concentrations (0.39–200 μg/mL). The reaction was carried out in a total volume of 100 μL. Quercetin was used as a reference inhibitor at final concentrations of 2, 5, 10, and 20 μL. After a 5 min reaction at 37 °C, the urea concentration was quantified using the UREA CE kit (Labtest^®^, Lagoa Santa, MG, Brazil) by spectrophotometry at 600 nm. The IC_50_ value was determined through a regression analysis of the dose–response curves.

#### 2.7.4. Reactive Oxygen Species (ROS) Assay

Promastigote forms at a final density of 2 × 10^7^ parasites/mL were treated, and the number of viable parasites was normalized as described in Section 2.7.2. The levels of reactive oxygen species (ROS) were determined by adding 20 μM H2DCFDA, a probe that emits fluorescence in the presence of ROS due to the formation of dichlorofluorescein (DCF) [39]. The fluorescence intensity was measured at 488/530 nm (excitation/emission) (SpectraMax i3x, Molecular Devices, Sunnyvale, CA, USA). A positive control of ROS production was performed using parasites exposed to 1 mM AAPH. Untreated parasites were used as negative controls.

#### 2.7.5. Autophagy Assay

Promastigote forms at a final density of 2 × 10^7^ parasites/mL were treated, and the number of viable parasites was normalized as described in Section 2.7.2 in a final volume of 100 µL. Following the incubation period, parasites were exposed to 100 µM MDA. After 1 h of incubation at 28 °C in the dark, the parasites were washed with PBS and fixed using 2% formaldehyde in saline solution. Fluorescence intensity was then measured at 335/460 nm (excitation/emission) using a SpectraMax i3x reader (Molecular Devices, Sunnyvale, CA, USA) [29]. Untreated parasites were used as negative controls.

### 2.8. Cytotoxicity Assay

The cytotoxicity of geopropolis extracts was assessed using various mammalian cell lines. RAW 264.7 and VERO cells were harvested at sub-confluence through trypsinization (10 min exposure to 0.25% trypsin in PBS), counted with a Neubauer chamber, and resuspended in complete DMEM at a final concentration of 10^6^ cells/mL. The cells were seeded into 96-well microplates (10^5^ cells/100 µL) and allowed to adhere for 1 h. Microplates containing MØ (10^5^ cells/100 µL) were obtained as described above. The cell cultures were treated with different concentrations of geopropolis extracts (15.6, 31.2, 62.5, 125, 250, 500, and 1000 µg/mL) for 48 h in a 5% CO_2_ atmosphere. After treatment, the cells were washed with PBS and incubated with MTT solution (1 mg/mL) for 4 h. The supernatant was then replaced with 100 µL of DMSO to solubilize the formazan crystals [40]. Absorbance was measured at 570 nm using a microplate reader (SpectraMax i3x, Molecular Devices, Sunnyvale, CA, USA). Each extract’s 50% cytotoxic concentration (CC_50_) was calculated using a nonlinear regression analysis of the dose–response curves.

The hemolytic potential of geopropolis extracts was evaluated using erythrocytes from *Ovis aries* (sheep). Blood samples purchased from *EBE Pharma Biológica e Agropecuária* (ethical approval 061/22-CEUA/UFRJ) were washed by centrifugation (1500× *g*/5 min) with PBS. A 4% (*v*/*v*) erythrocyte suspension was prepared, and 80 µL aliquots were transferred to a 96-well microplate containing 20 µL of geopropolis extracts (15 to 1000 µg/mL). The cells were incubated at 37 °C for 1 h, followed by adding 200 µL of PBS. Positive and negative controls for 100% and 0% hemolysis were prepared using distilled water or PBS, respectively, on untreated cells. The microplates were then centrifuged at 1700× *g*/10 min (centrifuge CT-6000, CIENTEC, Belo Horizonte, MG, Brazil), and 100 µL of supernatant from each well was transferred to a new microplate. Hemoglobin release was measured at 540 nm using a microplate reader (SpectraMax i3x, Molecular Devices, Sunnyvale, CA, USA) [40]. The 50% hemolytic concentration (CH_50_) was determined using a nonlinear regression analysis of the dose–response curves.

### 2.9. Macrophage Stimulation Assay

The effects of propolis extracts on NO production by MØ and RAW 264.7 macrophages were evaluated. Initially, uninfected macrophages were seeded and treated with the geopropolis extracts into 96-well microplates as described in Section 2.8 in a final volume of 100 µL, but with a slight modification. Concentrations below the CC_50_ (3.12, 6.25, 12.5, 25, 50, and 100 μg/mL) were used to avoid potential cytotoxicity. After incubation, culture supernatants (50 μL) were collected and distributed in a 96-well microplate. An equal volume of Griess reagent (0.5% sulfanilamide and 0.05% naphthyl ethylenediamine dihydrochloride in 5% phosphoric acid) was added. The reaction was incubated at room temperature for 20 min in the dark, and absorbance was measured at 570 nm. NO production was determined based on a sodium nitrite-calibration curve (0.195–100 µM) [41].

### 2.10. Statistical Analysis

All biological assays and experiments measuring total bioactive content and antioxidant capacity were performed in triplicate and repeated independently three times. Data normality was assessed using the Shapiro–Wilk test. Differences between the mean values of experimental groups were analyzed using either Student’s *t*-test or Tukey’s test (ANOVA) with a 95% confidence interval. A *p*-value < 0.05 was considered statistically significant. The statistical analyses were conducted using GraphPad Prism 8 software (GraphPad Software, Boston, MA, USA).

## 3. Results

### 3.1. Total Phenolic and Flavonoid Contents and Antioxidant Capacity

Table 1 presents the TPC of the extracts, which varied significantly (*p* < 0.05) among the samples. The MRGT extract exhibited the highest TPC (762 mg GAE/g), followed by the MDRY extract (528 mg GAE/g). The lowest TPC values were observed for the MNDA(1) and MNDA(2) extracts, at 105 and 126 mg GAE/g, respectively. Total flavonoid content (TFC) also varied across the samples, ranging from 23 to 344 mg QE/g. The MRGT extract displayed the highest TFC (344 mg QE/g), while the BCLR and MDRY extracts displayed TFC values of 113 and 315 mg QE/g, respectively. The lowest TFC values were observed in the MNDA(1) and MNDA(2) extracts, with 23 and 76 mg QE/g, respectively.

The antioxidant capacity of the extracts, evaluated using the DPPH method, revealed significant differences among the samples. The MRGT extract exhibited the highest antioxidant activity (56 ± 1.4%), significantly surpassing all other samples. The BCRL extract followed with an activity of 49 ± 0.1%, statistically distinct from the other extracts. Among the Melipona extracts, MNDY showed the lowest activity (25 ± 1.0%), while MNDA(1) and MNDA(2) exhibited intermediate values of 29 ± 0.8% and 31 ± 1.0%, respectively, with overlapping statistical groups. These results highlight the variability in antioxidant capacity across the different extracts.

### 3.2. Fingerprinting and Chemometric Analysis

**Figure 1:**
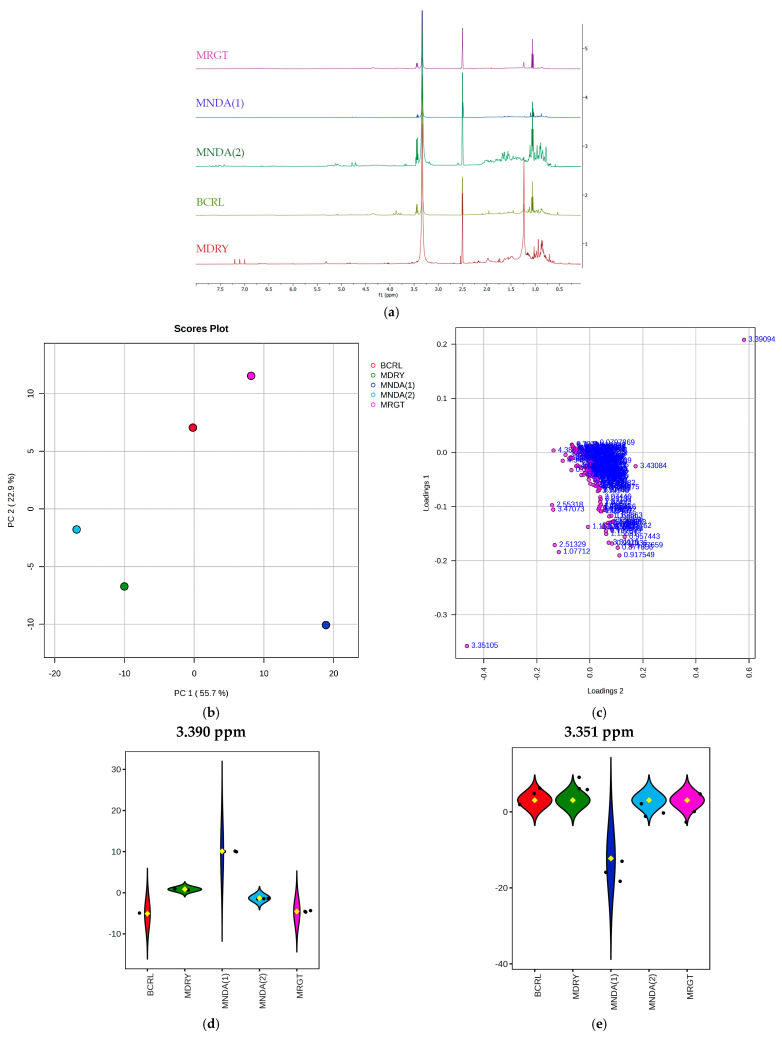
Spectral profiles and chemometric analysis of geopropolis extracts. (**a**) One-dimensional ^1^H NMR spectra of geopropolis extracts; (**b**) Principal Component Analysis (PCA) plot, showing the grouping of extracts based on their chemical profiles; (**c**) loadings plot highlighting the spectral regions (0.04 ppm buckets) that contribute most significantly to the differentiation of geopropolis extracts; (**d**,**e**) key spectral regions in the lower left (3.351 ppm) and upper right (3.390 ppm) identified as the primary contributors to the extracts’ discrimination. BCLR: geopropolis extract from *Melipona bicolor*; MDRY: geopropolis extract from *M. mondury*; MNDA(1): geopropolis extract from *M. quadrifasciata*; MNDA(2): geopropolis extract from *M. quadrifasciata*; MRGT: geopropolis extract from *M. marginata*.

The 1D ^1^H NMR spectra generated for the geopropolis extracts are shown in Figure 1a. The alignment of the spectra reveals a high degree of chemical similarity among the extracts represented by the signals at the same chemical shifts. All spectra share a major singlet at 3.33 ppm and a second at 2.5 ppm. However, unique features differentiate certain extracts. MNDA(2) exhibits signals between 4.5 and 5.5 ppm and near 7.5 ppm, which are absent in the other extracts. The MDRY extract shows three distinct singlets at 6.87, 7.08, and 7.19 ppm and a high-intensity signal at 1.23 ppm, which was not observed in the other samples. Conversely, the MNDA(1) extract displays lower signal intensities, particularly in regions between 0.5 and 2.9 ppm. Notably, the spectra were compared with those from other propolis studies, as no NMR data specific to stingless bee geopropolis was available. Unfortunately, the spectra of all extracts did not match any of the referenced studies, making it impossible to assign specific compounds. Nonetheless, the chemometric approach enabled an analysis of the chemical profile based on spectral signal intensities. Figure 1b presents the PCA, comparing the signal intensities of the 0.04 ppm buckets generated from the spectra of the different extracts. The results reveal a close similarity between the MDRY and MNDA(2) extracts, reflected in their proximity in the upper-left quadrant. Similarly, the BCLR and MRGT extracts are positioned in the lower-left quadrant of the graph. In turn, the MNDA(1) extract stands out, with bucket regions distinct from the other samples, appearing isolated in the upper-right corner of the PCA plot. The loading plot analysis in Figure 1c highlights the key signals (bucket regions) that contribute most to sample differentiation. Among these, the signals at 3.351 ppm and 3.390 ppm were the most significant for distinguishing the samples, as shown in Figure 1d,e.

The extracts were grouped using a heatmap clustering approach (Figure S1), which visualizes chemical similarity based on signal intensity, with colors ranging from blue (low intensity) to red (high intensity). As indicated by the PCA, MDRY, and MNDA(2) clustered together, as did BCLR and MRGT, reflecting their similar chemical profiles. In contrast, the MNDA(1) extract stood out with predominantly blue signals, indicating lower intensity, yet retained some chemical resemblance to the BCLR and MRGT cluster.

### 3.3. Anti-L. amazonensis Activity

All geopropolis extracts exhibited inhibitory activity against both promastigote and axenic amastigote forms of *L. amazonensis*. The IC_50_ values were determined after 48 h of exposure to the extracts (Table 2). Statistical analysis revealed no significant differences (*p* > 0.05) in anti-promastigote activity among MNDA(1), MNDA(2), and MRGT, with calculated IC_50_ values of 337 (y = −0.3043x + 150.89; r^2^ = 0.9755), 327 (y = −0.2695x + 134.41; r^2^ = 0.9745), and 339 (y = 159.59e^−0.003x^; r^2^ = 0.9934) µg/mL, respectively. In contrast, BCLR and MDRY extracts exhibited stronger activity, with IC_50_ values of 211 (y = −0.1228x + 77.091; r^2^ = 0.9858) and 154 (y = −89.028e^−0.004x^; r^2^ = 0.9433) µg/mL, respectively. The reference drug, SbIII, showed an IC_50_ of 144 µg/mL (y = −0.4488x + 114.72; r^2^ = 0.9368).

Notably, axenic amastigotes were more sensitive to the extracts than promastigotes, as reflected by lower IC_50_ values across all samples. MDRY showed the most potent anti-amastigote activity with an IC_50_ value of 20 µg/mL (y = −26.43In(x) + 128.22; r^2^ = 0.9413), followed by MNDA(1), which, despite its weaker activity against promastigotes, exhibited improved efficacy against amastigotes (IC_50_ = 22 µg/mL; y = 120.67e^−0.04x^; r^2^ = 0.9178). The remaining extracts BCRL, MNDA(2), and MRGT displayed IC50 values of 80 (y = −0.1211x + 100.26; r^2^ = 0.9161), 75 (y = −0.2533x + 160.06; r^2^ = 0.8845), and 81 (y = −81.08In(x) + 574.1; r^2^ = 0.9379) µg/mL. Similarly, SbIII showed an IC_50_ value of 70 µg/mL (y = -20.44In(x) + 149.21; r^2^ = 0.9824).

**Figure 2:**
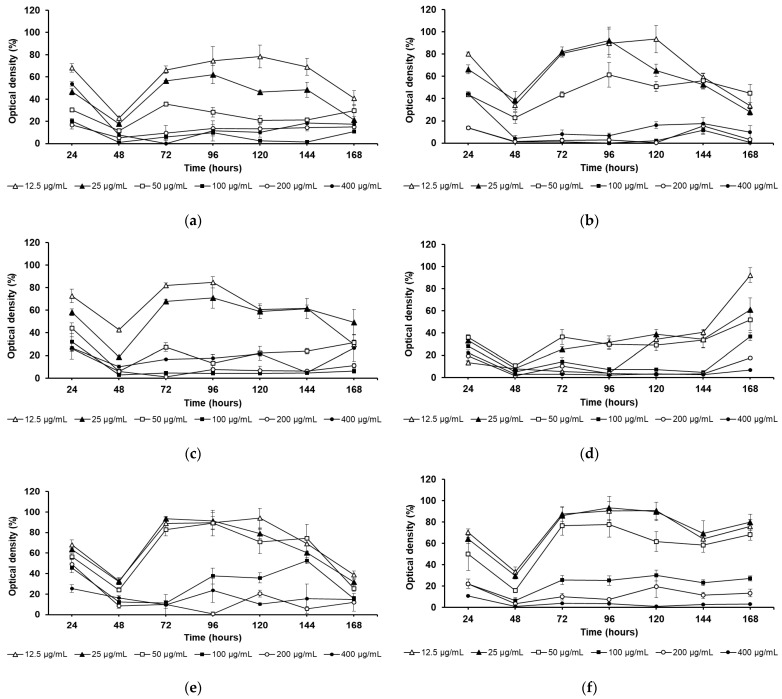
The effect of geopropolis extracts on the growth of *L. amazonensis* promastigotes. (**a**) Parasites treated with BCLR extract; (**b**) Parasites treated with MDRY extract; (**c**) Parasites treated with MNDA(1) extract; (**d**) Parasites treated with MNDA(2) extract; (**e**) Parasites treated with MRGT extract; and (**f**) Parasites treated with SbIII (reference drug). BCLR: geopropolis extract from *Melipona bicolor*; MDRY: geopropolis extract from *M. mondury*; MNDA(1): geopropolis extract from *M. quadrifasciata*; MNDA(2): geopropolis extract from *M. quadrifasciata;* MRGT: geopropolis extract from *M. marginata*; and SbIII: antimony potassium tartrate trihydrate. The experiments were performed in triplicate, and the results are expressed as mean ± standard error.

The growth curves of promastigotes treated with geopropolis extracts and the reference drug were monitored over 168 h to distinguish between leishmanistatic and leishmanicidal effects (Figure 2). At the 48 h mark, all extracts inhibited cell growth, but their effects diverged thereafter, showing distinct patterns. The MNDA(1) extract exhibited a leishmanicidal effect at 50 to 400 µg/mL concentrations, while growth was noted at 25 and 12.5 µg/mL concentrations after 48 h of treatment (leishmanistatic effect). The growth profile was similar when the parasites were treated with the BCLR and MNDA(1). MRGT displayed a leishmanicidal effect at 200 and 400 µg/mL. At a 100 µg/mL concentration, slight cell growth was observed between 72 and 144 h, followed by a decline at 168 h of treatment. MDRY showed a leishmanicidal effect at 100 to 400 µg/mL concentrations, inhibiting cell growth, while the other tested concentrations were leishmanistatic. The MNDA(2) extract had the greatest effect on cell growth among the extracts, as growth remained below 50% after 144 h. This extract displayed a leishmanicidal effect at 100 to 400 µg/mL. At 50 µg/mL, a leishmanistatic effect was observed from 96 h, while the other concentrations showed this effect starting at 48 h. SbIII exhibited a leishmanicidal effect at 200 and 400 µg/mL, while cell growth was observed between 12.5 and 50 µg/mL after the 48 h mark.

### 3.4. Mitochondrial Dehydrogenase Inhibition

**Figure 3:**
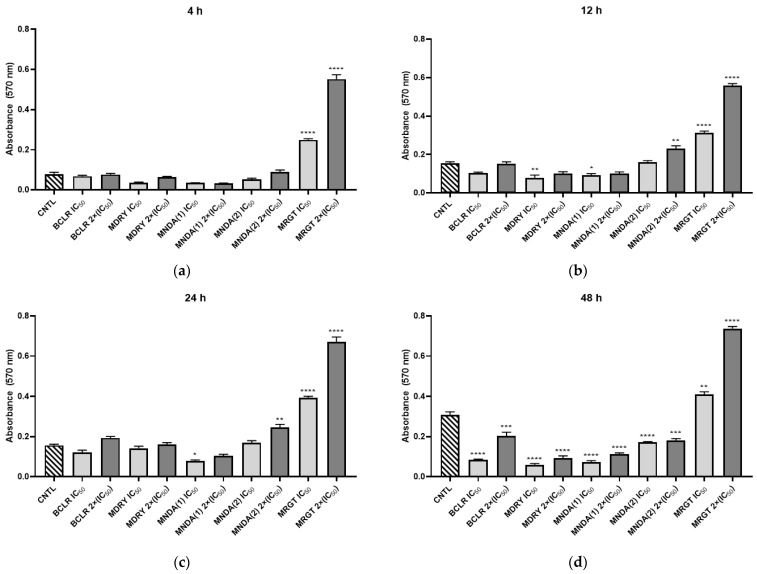
The effects of treatment with geopropolis extracts on mitochondrial dehydrogenases in *L. amazonensis* promastigotes after treatment with the extracts at concentrations corresponding to the IC_50_ and 2×(IC_50_). (**a**) Parasites treated with geopropolis extracts for 4 h; (**b**) Parasites treated with geopropolis extracts for 12 h; (**c**) Parasites treated with geopropolis extracts for 24 h; and (**d**) Parasites treated with geopropolis extracts for 48 h. CNTL: negative control (untreated); BCLR: geopropolis extract from *Melipona bicolor*; MDRY: geopropolis extract from *M. mondury*; MNDA(1): geopropolis extract from *M. quadrifasciata*; MNDA(2): geopropolis extract from *M. quadrifasciata;* MRGT: geopropolis extract from *M. marginata*. The bars in the graphs represent the mean values derived from two independent experiments, each performed in triplicate. Statistical analysis was performed using one-way ANOVA followed by Tukey’s post-test, comparing each treatment group with the control (untreated cultures). Significance levels are denoted as follows: * *p* < 0.05, ** *p* < 0.01, *** *p* < 0.001 and **** *p* < 0.0001.

Mitochondrial dehydrogenase inhibition was evaluated using geopropolis extracts at IC_50_ and 2×IC_50_ concentrations (Figure 3). After 4 h, parasites treated with BCRL, MDRY, MNDA(1), and MNDA(2) showed no significant changes in mitochondrial dehydrogenase activity compared to the control, regardless of the concentration tested. In contrast, the MRGT extract significantly increased enzymatic activity (*p* < 0.05) at both IC_50_ and 2×IC_50_ concentrations, and this effect persisted across all evaluated time points. At 12 h, MDRY and MNDA(1) displayed significant inhibitory effects at IC_50_ concentrations. By 24 h, only parasites exposed to MNDA(1) at the IC_50_ concentration exhibited significant inhibition of enzymatic activity (*p* < 0.05). Notably, parasites treated with MNDA(2) at 2×IC_50_ concentration showed a significant increase in enzymatic activity after 12 h and 24 h of exposure. At 48 h, all extracts—except for MRGT—demonstrated a significant inhibitory effect on mitochondrial dehydrogenase activity.

### 3.5. Inhibition of LiARG

**Figure 4:**
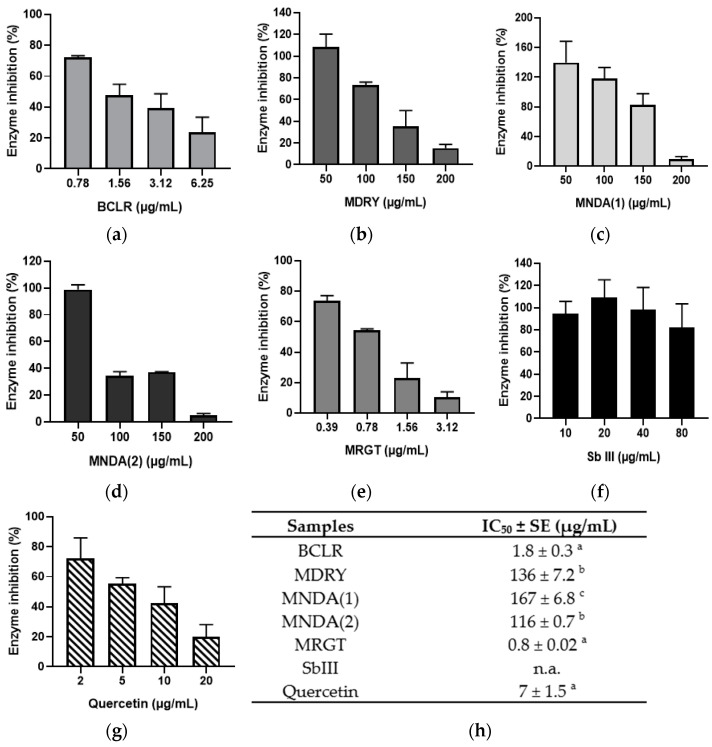
*Li*ARG inhibition activity of geopropolis extracts. Concentration–response bar graph of enzyme inhibition by (**a**) BCLR; (**b**) MDRY; (**c**) MNDA(1); (**d**) MNDA(2); (**e**) MRGT; (**f**) SbIII; and (**g**) quercetin. (**h**) Half-maximum enzyme inhibition activities of geopropolis extracts, SbIII, and quercetin. BCLR: geopropolis extract from *Melipona bicolor*; MDRY: geopropolis extract from *M. mondury*; MNDA(1): geopropolis extract from *M. quadrifasciata*; MNDA(2): geopropolis extract from *M. quadrifasciata;* MRGT: geopropolis extract from *M. marginata*; SbIII: antimony potassium tartrate trihydrate; n.a.: not active. The bars in the graphs and the values in the table represent the mean values ± standard error obtained from three independent experiments, with each experiment being conducted in triplicate. Different letters in (**h**) indicate significant differences (*p* < 0.05) between samples through statistical analysis using one-way ANOVA with Tukey’s multiple-comparisons test.

The inhibitory effects of geopropolis extracts on *Li*ARG and the reference drug SbIII and quercetin were evaluated (Figure 4). Among the extracts, MRGT exhibited the highest activity (IC_50_ = 0.8 µg/mL), followed by BCLR (IC_50_ = 1.8 µg/mL). MNDA(2) and MDRY showed IC_50_ values of 116 and 136 µg/mL, respectively, while MNDA(1) had the lowest activity (IC_50_ = 167 µg/mL). SbIII did not exhibit enzyme inhibition even at the highest concentration tested, whereas quercetin (reference inhibitor) showed an IC_50_ of 7 µg/mL. The inhibition percentages exhibited a concentration-dependent pattern.

### 3.6. Intracellular ROS Production by L. amazonensis

**Figure 5:**
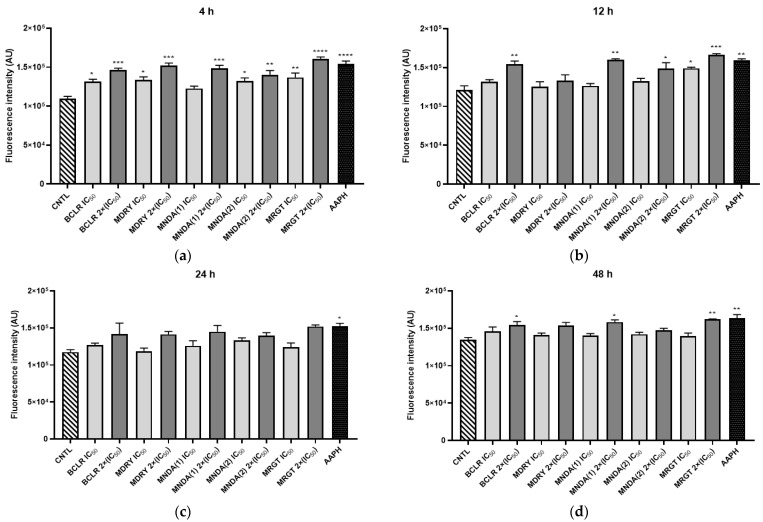
Effect of geopropolis treatment on intracellular ROS production in *L. amazonensis*. Promastigotes were treated with IC_50_ or 2×IC_50_ concentrations of each extract or with AAPH (1 mM) as a control. (**a**) Parasites treated with geopropolis extracts for 4 h; (**b**) Parasites treated with geopropolis extracts for 12 h; (**c**) Parasites treated with geopropolis extracts for 24 h; and (**d**) Parasites treated with geopropolis extracts for 48 h. CNTL: negative control (untreated); BCLR: geopropolis extract from *Melipona bicolor*; MDRY: geopropolis extract from *M. mondury*; MNDA(1): geopropolis extract from *M. quadrifasciata*; MNDA(2): geopropolis extract from *M. quadrifasciata;* MRGT: geopropolis extract from *M. marginata*; AAPH: Parasites exposed to 2,2′-azobis(2-methylpropionamidine) dihydrochloride (oxidative stress inducer). The bars in the graphs represent the mean values obtained from two independent experiments, with each experiment conducted in triplicate. Statistical analysis was performed using one-way ANOVA followed by Tukey’s post-test, comparing each treatment group with the control (untreated cultures). Significance levels are denoted as follows: * *p* < 0.05, ** *p* < 0.01, *** *p* < 0.001, and **** *p* < 0.0001.

Intracellular ROS production was determined after promastigote treatment with different geopropolis extracts at concentrations of IC_50_ and 2×(IC_50_) (Figure 5). At the fourth hour of incubation, ROS production increased for all systems tested compared to the control (untreated cells), except for MNDA(1) IC_50_. Notably, the highest induction of ROS was observed when the parasites were treated with 2×(IC_50_) extracts. At the 12 h mark, the highest induction of ROS was only observed at the extracts’ 2×(IC_50_) concentration. The exception was the MRGT extract, which, at IC_50_ concentration, was able to induce ROS with a significant difference (*p* < 0.05) from the untreated control. No significant statistical difference was observed between the extracts and the positive control (AAPH) on parasite oxidative stress. This result suggests that the extracts were as effective as AAPH in generating oxidative stress in the parasites. At 24 h, all concentrations of the tested extracts induced ROS compared to the untreated control. Finally, at 48 h, 2×(IC_50_) concentrations of BCRL, MNDA(2), and MRGT significantly induced ROS (*p* < 0.05) compared to the untreated parasites and similarly to AAPH.

### 3.7. Autophagic Activity

**Figure 6:**
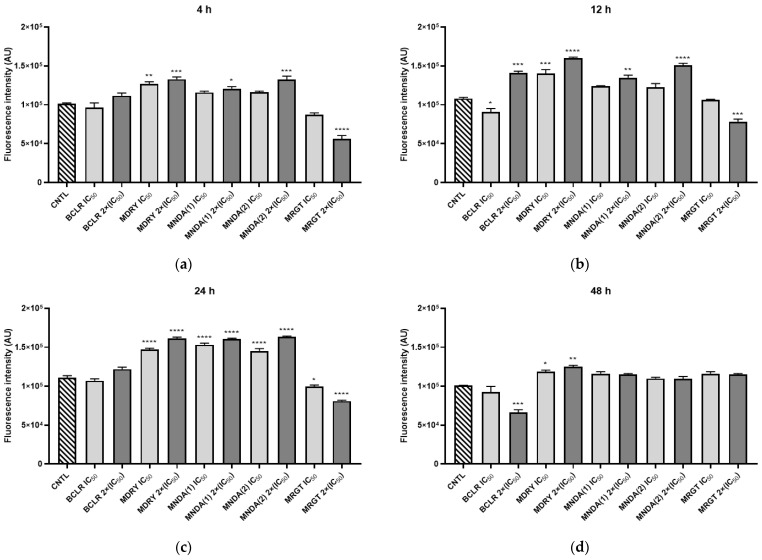
Effect of geopropolis extracts on autophagic activity in *L. amazonensis*. Promastigotes were treated with IC_50_ or 2×(IC_50_) concentrations of each extract. Non-treated parasites were used as controls. (**a**) Parasites treated with geopropolis extracts for 4 h; (**b**) Parasites treated with geopropolis extracts for 12 h; (**c**) Parasites treated with geopropolis extracts for 24 h; and (**d**) Parasites treated with geopropolis extracts for 48 h. CNTL: negative control (untreated); BCLR: geopropolis extract from *Melipona bicolor*; MDRY: geopropolis extract from *M. mondury*; MNDA(1): geopropolis extract from *M. quadrifasciata*; MNDA(2): geopropolis extract from *M. quadrifasciata;* MRGT: geopropolis extract from *M. marginata*. The bars in the graphs represent the mean values obtained from two independent experiments, with each experiment conducted in triplicate. Statistical analysis was performed using one-way ANOVA followed by Tukey’s post-test, comparing each treatment group with the control (untreated cultures). Significance levels are as follows: * *p* < 0.05, ** *p* < 0.01, *** *p* < 0.001, and **** *p* < 0.0001. Therefore, no comparison was performed in this case, as the purpose was to assess the relative impact of the treatments compared to the untreated control.

Promastigotes treated with geopropolis extracts at IC_50_ and 2×IC_50_ concentrations were assessed for autophagic activity (Figure 6). After 4 h of treatment, MNDA(1) and MNDA(2) at 2×(IC_50_), as well as MDRY at both IC_50_ and 2×(IC_50_), significantly increased autophagic activity (*p* < 0.05) compared to untreated parasites. In turn, MRGT and BLCR did not enhance the autophagic activity beyond that of the control parasites. Similar results were observed after 12 h of treatment. By 24 h, MRGT and BLCR did not enhance autophagic activity beyond that of the control parasites, while all other extracts significantly increased activity (*p* < 0.0001) regardless of the concentration. At 48 h, autophagic activity in treated parasites remained higher than in controls, with a statistical difference only for the MDRY extract at both concentrations.

### 3.8. Cytotoxic Potential of Geopropolis Extracts

The cytotoxic potential of geopropolis extracts was evaluated against different mammalian cell types (Table 2). The extracts showed moderate cytotoxicity against MØ, with CC_50_ values ranging from 201 (BCLR) to 644 (MDRY) µg/mL, while SbIII exhibited a CC_50_ of 104 µg/mL. Due to limited quantities of the MRGT extract, its cytotoxicity against MØ could not be assessed. RAW 264.7 cells were less sensitive, with CC_50_ values ranging from 417 BCLR to 673 (MRGT) µg/mL; SbIII had a CC_50_ of 128 µg/mL. For VERO cells, CC_50_ values ranged from 240 (MNDA(1)) to 684 (MRGT) µg/mL. MNDA(2) and BCLR also exhibited moderate cytotoxicity, with CC_50_ values of 296 µg/mL and 425 µg/mL, respectively. SbIII showed the highest toxicity against these cells, with a CC_50_ of 61 µg/mL. Sheep erythrocytes were the least sensitive to MNDA(2) and MDRY, with CC_50_ values of 710 µg/mL and 663 µg/mL, respectively. BCLR was the most hemolytic extract (CC_50_ = 254 µg/mL), while no hemolytic activity was observed for SbIII at the highest concentration tested (HC_50_ > 400 µg/mL). MRGT and MDRY were the least cytotoxic extracts, while BCLR showed the highest cytotoxicity for most cell types except VERO cells.

### 3.9. Effect of Geopropolis Extract Treatment on Uninfected Peritoneal Macrophages

**Figure 7:**
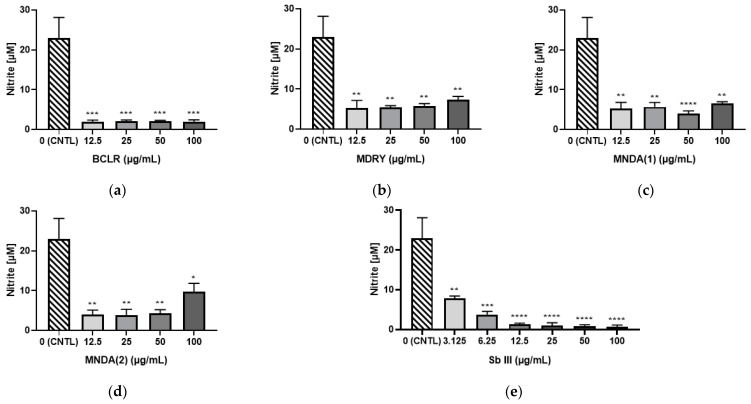
Effects of geopropolis extract treatment on non-infected peritoneal macrophages. (**a**) BCLR: geopropolis extract from *Melipona bicolor*; (**b**) MDRY: geopropolis extract from *M. mondury*; (**c**) MNDA(1): geopropolis extract from *M. quadrifasciata*; (**d**) MNDA(2): geopropolis extract from *M. quadrifasciata;* (**e**) Sb III: antimony potassium tartrate trihydrate. The bars in the graphs represent the mean values obtained from two independent experiments, with each experiment conducted in duplicate. Statistical analysis was performed using one-way ANOVA followed by Tukey’s post-test, comparing each treatment group with the control (untreated cultures). Significance levels are as follows: * *p* < 0.05, ** *p* < 0.01, *** *p* < 0.001, and **** *p* < 0.0001.

Figure 7 depicts the nitric oxide (NO) production by thioglycollate-elicited macrophages (MØ) treated or not treated with geopropolis extracts. All extracts significantly reduced (*p* < 0.05) nitrite levels compared to the untreated control, irrespective of the concentration used. Notably, only concentrations below the CC50 value for the evaluated cell type were considered, to ensure that cytotoxic activity did not influence the observed effects.

## 4. Discussion

Phenolic compounds, including flavonoids, are key bioactive classes present in various types of propolis and geopropolis, regardless of the producing bee species. Different countries have established minimum requirements for these compounds, with phenolic content ranging from 0.5% (m/m) in Brazil to 5% (m/m) in Argentina and Mexico. For flavonoids, the thresholds range from 0.25% in Brazil to 25% in Ukraine, ensuring the health benefits of *A. mellifera* propolis for human consumption [42,43]. High levels of phenolic compounds, measured as total phenolics and flavonoids, were observed across all geopropolis samples, with significant variation in their concentrations. This variability in phenolic and flavonoid content among propolis and geopropolis samples has been widely reported [24,27,44]. Notably, MNDA(1) and MNDA(2) had the lowest values, consistent with previous findings on *M. quadrifasciata* geopropolis [44]. NMR-based fingerprinting of the extracts revealed chemical similarities despite variations in signal intensities and unique signals in some samples. These differences were highlighted through the chemometric analysis. Similarly, the NMR spectra of *A. mellifera* propolis samples collected in different seasons showed more pronounced differences in the anomeric (4.50–5.50 ppm) and aromatic (5.50–8.50 ppm) regions of the spectra [45]. Bee species, botanical sources, and environmental conditions influence such variations. These factors are critical determinants of the bioactivity of geopropolis, including its antioxidant, antimicrobial, and anti-inflammatory effects [46,47].

MRGT demonstrated the highest antioxidant capacity, as evidenced by its superior DPPH^•^ scavenging activity at 0.25 mg/mL (Table 1). Overall, the antioxidant capacity observed here was higher than that reported for the stingless bee *Tetragonisca angustula* propolis, which exhibited a radical scavenging activity of 30.54% at 15 mg/mL [48]. Ferreira et al. (2024) showed that propolis produced by *Scaptotrigona postica* displayed a DPPH^•^ scavenging activity of 63.95% at 2 mg/mL [49]. In a previous study, *M. mondury* geopropolis demonstrated an even more potent antioxidant capacity, inhibiting 50% of DPPH^•^ at 6.91 µg/mL [27]. As mentioned earlier, the composition of propolis and geopropolis is influenced by various factors, including bee species and environmental conditions, which collectively can impact their antioxidant potential. This scenario highlights the importance of standardized methods for extracting propolis and geopropolis to enable more consistent and comparable evaluations of their health benefits [46].

The antioxidant property is particularly relevant in the context of tegumentary leishmaniasis, characterized by a Th1-mediated pro-inflammatory response. Exacerbating this inflammatory process creates an oxidative and nitrosative environment, leading to tissue damage and, in severe cases, significant lesions and disfigurement [50,51]. The observed reduction in NO production by thioglycolate-stimulated macrophages (Figure 7) highlights the anti-inflammatory potential of geopropolis, aligning with the known properties of propolis [52,53]. A previous study demonstrated that the 4-phenyl coumarin cinnamoyloxy-mammeisin, isolated from *Melipona scutellaris* geopropolis, is responsible for its anti-inflammatory activity by inhibiting the MAPK signaling, AP-1, and NF-κB pathways [54]. The antioxidant and anti-inflammatory properties of stingless bee geopropolis may help mitigate the exacerbated pro-inflammatory response to *Leishmania*, potentially preventing tissue damage and lesion progression, as demonstrated by other natural products [55]. It is important to note that while nitric oxide (NO) production is a key antileishmanial mechanism employed by infected macrophages, several studies have shown that natural products, particularly phenolic compounds, can eliminate parasites within macrophages through NO-independent pathways [56,57].

Few studies have explored the activity of Brazilian geopropolis against *Leishmania* spp. Dutra et al. (2019) reported that the geopropolis from *Melipona fasciculata* exhibits anti-*L. amazonensis* activity, associating its effect with major compounds such as gallic and ellagic acids. The hydroalcoholic crude extract demonstrated an IC_50_ of 47 µg/mL against promastigotes [58]. In contrast, *L. amazonensis* promastigotes were less sensitive to *Scaptotrigona postica* propolis, with more than 50% viability observed even at the highest concentration tested (500 µg/mL) [49]. In the present study, while *L. amazonensis* promastigotes exhibited higher IC_50_ values for the tested geopropolis extracts, axenic amastigotes were notably more sensitive, particularly to MDRY and MNDA(1), which were statistically more effective than SbIII. These findings align with the broader recognition of various bee propolis types for their antiparasitic activity [59,60,61]. Additionally, a leishmanistatic effect was observed primarily at 50 and 100 µg/mL for most extracts, except for MNDA(2), which exhibited leishmanistatic activity across a broader range. Although leishmanicidal concentrations are often preferred, leishmanistatic concentrations also offer advantages, as they can complement more potent leishmanicidal drugs. In fact, combination therapies using leishmanicidal drugs alongside the leishmanistatic oral drug allopurinol have shown high efficacy in treating canine visceral leishmaniasis [62,63]. Here, geopropolis extracts demonstrated promising antileishmanial activity, including a leishmanistatic effect at lower concentrations. However, it is noteworthy that testing the geopropolis extracts against other *Leishmania* species is essential to gain further insights into their effectiveness as antileishmanial agents and their potential as a promising source of antileishmanial compounds.

Notably, the heightened sensitivity of axenic amastigotes to the extracts may be attributed to metabolic changes. During differentiation from promastigotes to axenic amastigotes, the parasite downregulates genes related to translation and ribosome biogenesis while upregulating the translation of amastigote-specific proteins, such as stress-response proteins [64]. Although these adaptations enhance amastigote survival within the hostile environment of the parasitophorous vacuole, they may also increase susceptibility to bioactive compounds. Furthermore, promastigotes, as the extracellular form, are adapted to survive in the midgut of the sandfly, an environment requiring greater resistance to external stressors [65]. In turn, amastigotes reside within the relatively protected environment of host macrophages, potentially making them less equipped to withstand certain stress-inducing compounds found in the extracts. These physiological and environmental differences likely contribute to the observed higher sensitivity of amastigotes to the tested geopropolis extracts. In this study, the mode of action of geopropolis extracts was evaluated against promastigote forms due to their ease of cultivation and the straightforward, reproducible nature of this model for preliminary screening, which ensures consistent results across multiple assays. Although amastigotes represent the medically relevant form, most studies in the literature begin by evaluating promastigote susceptibility, providing a comparative baseline for the findings presented here. Nonetheless, further studies are needed to evaluate the activity of geopropolis extracts against intracellular amastigote forms. Investigating the treatment of infected host cells could undoubtedly provide valuable insights into the potential efficacy of geopropolis extracts for leishmaniasis therapy [66].

Naturally occurring phenolic compounds are well documented for their antileishmanial activity [38,67,68], supporting our hypothesis that the pronounced activity of MDRY may be linked to its high TPC and TFC values. In contrast, MGRT, despite its high phenolic content, exhibited less pronounced activity, while MNDA(1) demonstrated a similar antileishmanial effect despite its lower phenolic levels. These results suggest that other chemical classes may trigger the antileishmanial activity. Cuesta-Rubio et al. (2017) reported the antileishmanial activity of three propolis extracts collected in Ecuador, finding high amounts of terpenoids in all samples [69]. However, the most active extract against amastigotes was rich in flavonoids, aligning with the results described here. Further investigation is needed to identify the specific compounds responsible for driving the antileishmanial effects of geopropolis extracts and explore potential synergistic interactions between their bioactive components.

The MTT assay is widely used to assess cell viability across various cell types. Although extramitochondrial MTT reduction has been reported [70], numerous studies support its reliability in evaluating mitochondrial dehydrogenase activity. The use of respiratory chain inhibitors, such as malonate (a succinate dehydrogenase inhibitor) [71] and rotenone (a mitochondrial NADH dehydrogenase inhibitor) [72], has further validated this assay for assessing mitochondrial dehydrogenase activity and has also facilitated the identification of novel inhibitors or enhancers of these enzymes. Unlike mammals, some plants, bacteria, fungi, and protozoa possess a distinct type of dehydrogenase known as type II NADH dehydrogenase (NDH2). This enzyme oxidizes NADH, regenerating NAD^+^ and contributing to ATP production, thereby playing a critical role in the energy metabolism of these organisms, including many pathogens. Duarte et al. (2021) demonstrated that *Leishmania* NDH2 is essential for parasite survival [73]. Additionally, natural products have been identified as promising inhibitors of *Leishmania major* cytosolic 1A DHODH (dihydroorotate dehydrogenase), a flavoenzyme in trypanosomatids. Among these, sesquiterpene lactones (glaucolide B and 2-Oxo-8β-tigloyloxy-guaia-1(10),3,11(13)-trien-6α,12-olide), a diterpene (*ent*-kaurenoic acid), and a flavonoid (myricetin) demonstrated significant inhibitory activity [74]. These results highlight dehydrogenases as promising targets for developing therapeutic drugs against infectious diseases. In the present study, MNDA(1) and MDRY inhibited *L. amazonensis* mitochondrial dehydrogenases throughout the treatment period, suggesting that disrupting these enzymes could play a role in eliminating the parasite. In a previous study, notably, to our knowledge, there is no prior evidence that propolis affects mitochondrial dehydrogenases in *Leishmania* parasites.

The Brazilian green propolis, a product of honeybees, demonstrated immunomodulatory effects that resulted in a reduction in macrophage infection. However, preliminary data suggest no ARG gene expression modulation was observed [75]. Despite this, previous studies have shown that naturally occurring phenolic compounds are potent direct inhibitors of *Leishmania* ARG [18,76,77]. Since geopropolis extracts are rich in these phenolic compounds, we expected to observe an inhibitory effect on the enzyme. Among the extracts, the phenolic-rich MRGT and BCLR displayed the strongest activity against *Li*ARG. Both extracts demonstrated higher inhibition levels than quercetin, a known *Li*ARG inhibitor. Interestingly, MNDA(2), despite having the lowest phenolic content, exhibited potent enzymatic inhibition, suggesting that other chemical classes may contribute to *Li*ARG inhibition. SbIII did not show LiARG inhibitory activity. However, previous research has demonstrated that SbIII inhibits trypanothione reductase (TR) activity, thereby disrupting T(SH)_2_ formation [78,79]. The inhibition of ARG by geopropolis extracts could have significant implications for parasite proliferation and differentiation, potentially increasing intracellular oxidative stress [80]. These effects may be particularly impactful when combined with TR inhibition in a synergistic therapy involving antimonials. Indeed, when reactive oxygen species (ROS) induction was evaluated, it was found that the geopropolis extracts enhanced ROS production in the parasite. This effect was particularly noticeable in systems treated with 2×(IC_50_), where ROS levels were comparable to those observed with the positive control (AAPH). Disrupting free radicals in the parasite can be an effective strategy for elimination, as they can cause structural damage or activate signaling pathways that lead to apoptosis [34]. Notably, MNDA(1), MNDA(2), and MDRY enhanced parasite autophagic activity after 48 h treatment. Autophagy can function as a protective mechanism, recycling macromolecules and removing damaged organelles, but excessive autophagic activity can be a sign of an apoptotic process, resulting in parasite death [81].

Overall, geopropolis cytotoxicity was moderate-to-low against mammalian cells, with BCLR exhibiting the highest toxicity for most cell types (MØ, RAW 264.7, and erythrocytes). At the same time, MNDA(1) displayed the highest cytotoxic activity against VERO cells. Considering that a selectivity index (SI) greater than 1 indicates that the drug is more selective for the parasite than for the host cell, the majority of extracts in this study reached this value. However, SI values greater than 10 are considered desirable for parasitic diseases, as they represent a lower toxicity risk for the host [82]. The MDRY stood out in this scenario, with SI values varying between 21 and 33. Additionally, MNDA(1) showed promise, with SI values for amastigotes greater than 10 for MØ, RAW 264.7 macrophages, the relevant host cells, and erythrocytes.

## 5. Conclusions

This study evaluated five different geopropolis extracts, with the MDRY extract demonstrating the most promising activity against both promastigotes and axenic amastigotes. The results underscore the MDRY extract potential as a source of antileishmanial agents. The extract’s high levels of phenolics, flavonoids, and antioxidant capacity could be key factors for controlling *L. amazonensis* infection. Overall, the extracts inhibited mitochondrial dehydrogenases and *Li*ARG and induced the production of reactive oxygen species and autophagic activity. These results offer insights into the potential mechanisms of action of geopropolis extracts. However, further studies are needed to investigate the interactions of MDRY and the other geopropolis extracts with intracellular amastigotes and to conduct in vivo assays to confirm their promising antileishmanial activity.

## Figures and Tables

**Table 1:** Total phenolic content (TPC), total flavonoid content (TFC), and antioxidant capacity of geopropolis extracts.

Samples	TPC (mg GAE/g)	TFC (mg QE/g)	DPPH^•^ (%)
BCLR	278 ± 18 ^b^	113 ± 4.8 ^a^	49 ± 0.1 ^a^
MDRY	528 ± 28 ^d^	318 ± 20 ^b^	25 ± 1.0 ^b^
MNDA(1)	126 ± 20 ^a^	76 ± 2.2 ^a^	29 ± 0.8 ^b,c^
MNDA(2)	105 ± 14 ^a^	23 ± 0.9 ^c^	31 ± 1.0 ^c^
MRGT	762 ± 31 ^c^	344 ± 16 ^b^	56 ± 1.4 ^d^

BCLR: geopropolis extract from *Melipona bicolor*; MDRY: geopropolis extract from *M. mondury*; MNDA(1): geopropolis extract from *M. quadrifasciata*; MNDA(2): geopropolis extract from *M. quadrifasciata;* MRGT: geopropolis extract from *M. marginata*. Results are expressed as mean ± standard error of three independent experiments carried out in triplicate. Statistical analysis was performed using one-way ANOVA with Tukey’s multiple-comparisons test to determine significant differences (*p* < 0.05) between means represented by different letters in each column.

**Table 2:** Antileishmanial activity and cytotoxicity of geopropolis extracts. The IC_50_ values for promastigotes and axenic amastigotes of *Leishmania amazonensis* were determined after 48 h of treatment.

Extract	*L. amazonensis*		MØ		RAW 264.7		VERO		ERY	
	IC_50/PRO_	IC_50/AMA_	CC_50_ ± SE	IS_AMA_	CC_50_ ± SE	IS_AMA_	CC_50_ ± SE	IS_AMA_	CC_50_ ± SE	IS_AMA_
BCLR	211 ± 18 ^b^	80 ± 16 ^b^	201 ± 24 ^b^	2.5	417 ± 18 ^a^	5.2	307 ± 6.4 ^b^	3.8	254 ± 24 ^a^	3.1
MDRY	154 ± 4.6 ^b^	20 ± 2.1 ^a^	644 ± 14 ^a^	32	481 ± 16 ^a^	24	425 ± 11 ^d^	21	663 ± 29 ^c^	33
MNDA(1)	337 ± 2.8 ^a^	22 ± 2.1 ^a^	580 ± 14 ^a^	26	419 ± 21 ^a^	19	240 ± 3.8 ^a^	11	402 ± 26 ^a^	18
MNDA(2)	327 ± 43 ^a^	75 ± 4.5 ^b^	220 ± 5.1 ^b^	2.9	432 ± 22 ^a^	5.8	296 ± 11 ^b^	3.9	710 ± 26 ^d^	9.5
MRGT	339 ± 34 ^a^	81 ± 12 ^b^	n.d.	n.d.	673 ± 58 ^b^	8.3	684 ± 9.1 ^c^	8.4	383 ± 13 ^a^	4.7
SbIII	144 ± 5.2 ^b^	70 ± 7.9 ^b^	104 ± 5.4 ^b^	1.5	128 ± 10 ^c^	1.8	61 ± 0.7 ^e^	0.9	>400	-

n.d. = not determined. BCLR: geopropolis extract from *Melipona bicolor*; MDRY: geopropolis extract from *M. mondury*; MNDA(1): geopropolis extract from *M. quadrifasciata*; MNDA(2): geopropolis extract from *M. quadrifasciata;* MRGT: geopropolis extract from *M. marginata*; SbIII: antimony potassium tartrate trihydrate; MØ: peritoneal macrophages; and ERY: erythrocytes. The selectivity index for amastigotes (SI_AMA_) was determined as CC_50_/IC_50_ ratio. The results are expressed as mean ± standard error of three independent experiments in triplicate. Statistical analysis was performed using one-way ANOVA with Tukey’s multiple-comparisons test to determine significant differences (*p* < 0.05) between means represented by different letters in each column.

## Data Availability

The data that support the findings of this study are available from the corresponding author upon reasonable request.

## Supplementary Materials

The following supporting information can be downloaded at: https://www.mdpi.com/article/10.3390/biology14020162/s1, Figure S1: A two-dimensional hierarchical clustering heatmap of the chemical profiles of geopropolis extracts.
