# Supplementary material for: Meliponini Geopropolis Extracts Induce ROS Production and Death in Leishmania amazonensis Promastigotes and Axenic Amastigotes In Vitro
Source: Biology (Basel). 2025 Feb 6;14(2):162. doi: 10.3390/biology14020162 (PMC11851448; doi:10.3390/biology14020162)
Supplement: Supplementary file 1 [file biology-14-00162-s001.zip › biology-3387309-supplementary.pdf]

biology

MDPI

Supplementary

Meliponine geopropolis induce oxidative stress and killing of Leishmania amazonensis

Kamila Sette [1,6], Andreza R. Garcia [1,6], Luzineide Tinoco [2], Anderson S. Pinheiro [3], Igor Rodrigues [4,*]

[1] Programa de Pós Graduação em Ciências Farmacêuticas, Faculdade de Farmácia, Universidade Federal do Rio de Janeiro, Rio de Janeiro 21941-902, , RJ, Brasil; kamila.sette@hotmail.com

[2] Laboratório Multiusuário de Análises por RMN, Instituto de Pesquisa de Produtos Naturais, Universidade Federal do Rio de Janeiro, Rio de Janeiro 21941-902, RJ, Brasil; luzitinoco@gmail.com

[3] Laboratório de Bioquímica Molecular, Departamento de Bioquímica, Instituto de Química, Universidade Federal do Rio de Janeiro, Rio de Janeiro 21941-902, RJ, Brasil; pinheiro@iq.ufrj.br

[4] Laboratório de Investigação de Substâncias Bioativas, Departamento de Produtos Naturais, Faculdade de Farmácia, Universidade Federal do Rio de Janeiro, Rio de Janeiro 21941-599, RJ, Brasil; igor@pharma.ufrj.br

* Correspondence: igor@pharma.ufrj.br

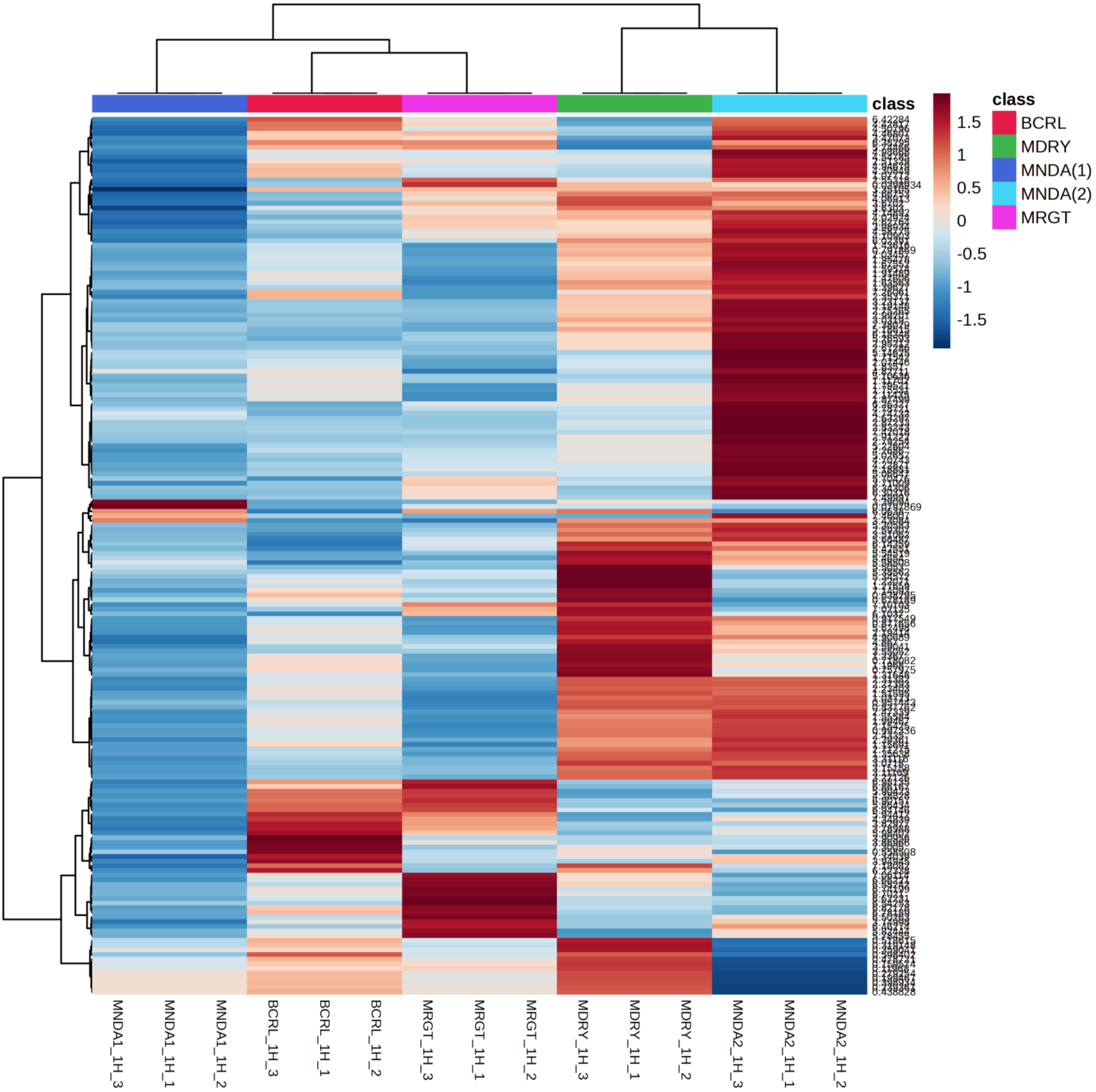

Figure S1. Two-dimensional hierarchical clustering heatmap of the chemical profiles of geopropolis extracts. This analysis visualizes the similarities and differences between samples based on the spectral intensities of bucketed regions (0.04 ppm). Abbreviations: MNDA – geopropolis extract from Melipona quadrifasciata; BCLR – geopropolis extract from Melipona bicolor; MRGT – geopropolis extract from Melipona marginata; MDRY – geopropolis extract from Melipona mondury; MBB – geopropolis extract from Melipona quadrifasciata.
